# Platelet-Rich Plasma in Fat Graft Type-1 Tympanoplasty – Should We or Should We Not?

**DOI:** 10.1055/s-0044-1787169

**Published:** 2024-06-04

**Authors:** Bikram Choudhury, Palak Gupta, Saptarshi Mandal, Vidhu Sharma, Kapil Soni, Darwin Kaushal

**Affiliations:** 1 Department of Ear, Nose, and Throat, All India Institute of Medical Sciences (AIIMS), Jodhpur, India; 2 Department of Transfusion Medicine and Blood Bank, All India Institute of Medical Sciences (AIIMS), Jodhpur, India; 3 Department of Ear, Nose, and Throat, All India Institute of medical sciences (AIIMS), Jammu, India

**Keywords:** platelet-rich plasma, tympanoplasty, suppurative otitis media, eardrum perforation

## Abstract

**Introduction** Fat grafts have been in used since 1962 for small central perforations, with a success rate ranging from 86 to 100%. Platelet-rich plasma (PRP) containing platelet concentrations greater than 1 million platelets/μL assist the healing process by various means. Current data suggests improved healing when tympanoplasty is performed using temporalis fascia grafts if PRP is added during surgery.

**Objective** To assess the effect of PRP on fat grafts in small and moderate-sized central perforations.

**Methods** The present prospective observational study was conducted with 36 patients who underwent fat graft tympanoplasty with PRP under local anesthesia. Clinical and audiological observations were carried out after 4, 8, and 12 weeks, and a statistical analysis of the observations was performed.

**Results** We assessed 23 patients with small central perforations and 11 patients with moderate central perforations. An overall success rate of 76.4% was observed, with an 82.6% success rate among patients with small central perforations and 63.6% among those with moderate central perforations. There was no statistically significant difference in the uptake regarding the location of the perforation, but a statistically significant difference was found in terms of hearing improvement following the procedure.

**Conclusion** The morbidity of conventional tympanoplasty in cases of small-to-moderate central perforations in patients with chronic otitis media vis a vis the results of the procedure needs to be revisited, as in the present study fat grafts placed with PRP under local anesthesia could lead to surgical and audiological outcomes that are as good as those reported in the literature.

## Introduction

Fat graft type-1 tympanoplasty has been in use since 1962 1 for the repair of small central perforations, with a success rate ranging from 86 to 100%. 2 In comparison, the temporalis fascia, which used to be the most commonly used graft material, 3 presented a success rate of up to 88% in a meta-analysis. 4 Fat grafts are a better alternative for the repair of small perforations because of their ability for neoangiogenesis, with postoperative shrinkage in size due to acute necrosis and subsequent generation of new microvessels. Platelet-rich plasma (PRP) is autologous plasma that has a platelet concentration of at least 1 million platelets/μL. 5 Platelet granules consist of bioactive molecules that, when activated, result in the proliferation and differentiation of cells with anabolic and proinflammatory properties, which favors the healing process.

The present study aimed to assess the effect of PRP on fat grafts when used in small and moderate-sized perforations in terms of graft uptake and hearing benefits.

## Methods

The present prospective observational study was conducted at a tertiary care center in the Department of Otorhinolaryngology of our institution between October 2018 and March 2020. The study was approved by the institutional Ethics Committee (AIIMS/IEC/2018/659), and informed consent was taken from the patients prior to their enrolment in the study. The study followed the guidelines of the Strengthening the Reporting of Observational Studies in Epidemiology (STROBE) statement. We included 36 patients suffering from chronic suppurative (mucosal) otitis media, with dry ears for more than two weeks without the use of medications. All patients with acute ear infection, those younger than 15 years of age, pregnant women, or patients who had any hematological condition making retrieval of PRP difficult were excluded. Moreover, patients with any pre- or intraoperative evidence of cholesteatoma, cases of revision surgery cases, and patients who required other types of tympanoplasty (types 2 to 5) were also excluded.

**Fig. 1:**
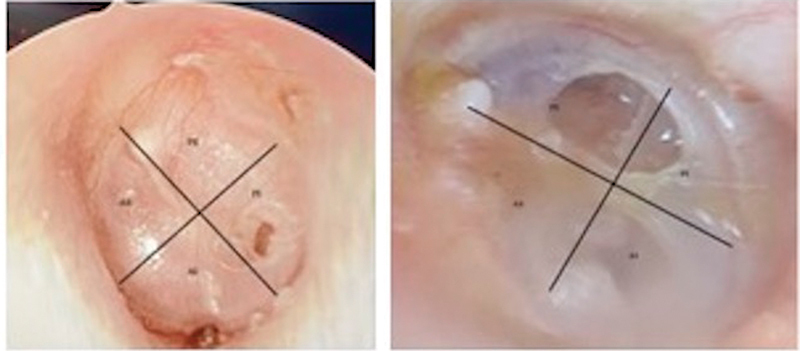
Small and moderate-sized central perforations.

Only small and moderate-sized perforations, classified according to the Saliba 6 method, were included ( Fig. 1 ). Pure-tone average audiometry and the air-bone gap were recorded over the frequencies of 500 Hz, 1,000 Hz, 2,000 Hz, and 4,000 Hz, pre- and postoperatively. The PRP was prepared using 10 mL of whole blood that was withdrawn from the subjects on the morning of the surgery. It was centrifuged initially at 1,500 rpm for 15 minutes (soft spin) to form the triple layer. The topmost layer was then separated in a fresh autoclaved tube and centrifuged again at 2,500 rpm for 10 minutes (hard spin). The PRP thus obtained was mixed and kept inside the platelet agitator for 30 minutes. Once ready, the PRP was transported to the operation theatre at the time of the procedure.

All procedures were performed under local anesthesia, via the transcanal approach; 0.5 mL of local anesthesia (lignocaine and adrenaline, at a ratio of 1:200,000), was infiltrated into the ipsilateral ear lobule and the ear canal at the bony cartilaginous junction at the 3, 6, 9, and 12'o clock positions. An incision of approximately 1.5 cm was made in the posterior aspect of the lobule, and an adequate amount of fat (at least 2 times the size of the perforation) was obtained and placed in normal saline. The margins of the perforation were freshened using sharp straight and curved picks. Two small pieces of Gelfoam (Pharmacia & Upjohn Company, LLC, Kalamazoo, MI, United States) were inserted into the middle ear cavity through the perforation, and 0.2 mL of PRP was instilled. Fat that had been harvested previously was inserted in the shape of a dumbbell, to lie equally medially and laterally to the perforation. The graft was secured in place with ciprofloxacin-coated (0.2%, 0.5 mg in 0.25 mL) Gelfoam pieces, and the PRP was instilled over it. All patients were observed for a few hours and then discharged on medications, and appropriate precautions were explained. We considered that graft uptake had occurred if, at the end of 3 months, the tympanic membrane with the graft in situ was mobile on pneumatic otoscopy. Graft uptake was considered successful if hearing improvements were observed along with a mobile tympanic membrane. The IBM SPSS Statistics for Windows (IBM Corp., Armonk, NY, United States) software, version 23.0, was used for the statistical analysis.

## Results

The mean age of the patients was 30.82 years; 23 (63.8%) subjects presented small and 13 (36.1%) presented moderate-sized central perforations. Of the 23 small perforations, 13 were located anteriorly, and 10, posteriorly. ( Table 1 ).

**Table 1:** Graft uptake results in terms of size, site of the perforation, and patient characteristics

Size of perforation	Male	Female	Total	Central	Anterior	Posterior
Small **: n (%)**	**11 (47.8%)**	**12 (52.1%)**	**23 (63.8%)**	**0 (0.0%)**	**13 (56.5%)**	**10 (43.4%)**
Moderate **: n (%)**	**4 (30.7%)**	**9 (69.2%)**	**13 (36.1%)**	**2 (15.3%)**	**6 (46.1%)**	**5 (38.4%)**

**Table:** 

		Size of the perforation		Total
		Moderate	Small	
Graft uptake result: n (%)	Success	7 (63.6%)	19 (82.6%)	26 (76.4%)
	Failure	4 (36.3%)	4 (17.3%)	8 (23.5%)
Total: n (%)		11 (32.3%)	23 (67.6%)	34 (100%)

**Table:** 

		Graft uptake results		Total
		Success	Failure	
Site of the perforation: n (%)	Anterior	14 (77.7%)	4 (22.2%)	18
	Posterior	11 (73.3%)	4 (22.2%)	15
Total		25	8	33*

**Table:** 

*Factor*		*N* *=* *34*	*Graft uptake success* : n *(%)*	*p-value*
*Age*	**< 40 years**	26	19 (73.0)	0.645
	**> 40 years**	8	7 (87.5)	
*Sex*	**Male**	15	12 (80.0)	1.00
	**Female**	19	14 (73.6)	
*Size*	**Small**	23	19 (82.6)	0.388
	**Moderate**	11	7 (63.6)	
Site*	**Anterior**	18	14 (77.7)	1.00
	**Posterior**	15	11 (73.3)	
*Side*	**Unilateral**	23	19 (82.0)	0.388
	**Bilateral**	11	7 (63.6)	

Note: *The perforation involved small regions of both anterior as well as posterior parts of the tympanic membrane. The perforation was present in the center of the membrane, and thus to avoid any discrepancies in the results, we excluded this case.

Two patients were lost to follow-up after the first visit, so their graft status could not be assessed. Out of the remaining 34 patients, 23 (67.6%) presented small, and 11 (32.3%) had moderate-sized perforations. An overall rate of successful graft uptake of 26 (76.4%) was observed: 19 (82.6%) patients with small perforations, and 7 (63.6%) with moderate-sized perforations. ( Table 1 ). Using the Fischer exact test, however, the findings were not statistically significant ( *p* = 0.388).

When the site of perforation was related with the success rate, 14 (77.7%) anterior and 11 (73.3%) posterior perforations presented successful graft uptake 3 months postoperatively ( Table 2 ), but this finding was not statistically significant ( *p* = 1.00). Table 2 also shows the relationships regarding different prognostic factors and the graft outcome.

**Table 2:** Pre- and p-ostoperative audiometry results

	Preoperative pure-tone average	Preoperative air-bone gap	Postoperative pure-tone average	Postoperative air-bone gap
**Mean ± standard deviation**	38.6333 ± 13.28334	23.8000 ± 7.71206	30.5667 ± 11.15621	15.7667 ± 5.81724
				

**Table:** 

	Mean ± standard deviation		*t* -value	*p*
**Difference in pure-tone average**	8.06667 ± 2.85190		15.492	< 0.001
**Difference in air-bone gap**	8.03333 ± 3.18924		13.797	< 0.001

The mean preoperative pure-tone average for 30 patients (after excluding 6 patients that were lost to follow up) was of 38.63 ± 13.2 dB, and the mean postoperative pure tone average was of 30.56 ± 11.15 dB. The mean pre and postoperative air-bone gaps were of 23.80 ± 7.7) dB and 15.76 ± 5.81) dB, respectively ( Table 2 ). The pre- and postoperative pure tone averages showed a mean difference of 8.06 ± 2.85 dB, which was statistically significant ( *p* < 0.001); regarding the air-bone gaps, the mean difference was of 8.03 ± 3.18 dB, which was also statistically significant ( *p* < 0.001) ( Table 2 ).

## Discussion

In the present study, the mean age of the patients was 30.82 years, with 26 (77.7%) younger than 40 years and 8 (23.3%) older than 40 years of age. This difference in terms of age did not result in any significant differences in the graft uptake results after 3 months of follow-up ( *p* = 0.645).

Ebrahim et al. 2 and Ersözlü and Gultekin 7 achieved an overall success rate of 85.7%, with 100% of success in small central perforations with fat graft and PRP type-1 tympanoplasty. In the present study, the success rate was 26 out of 34 (76.4%) in terms of graft uptake, with 82.6% (19 patients) of success in small perforations; among moderate-sized perforations, the rate was of 63.6% (7 subjects), in contrast to 79.3% in the study by Ebrahim et al. 2

**Fig. 2:**
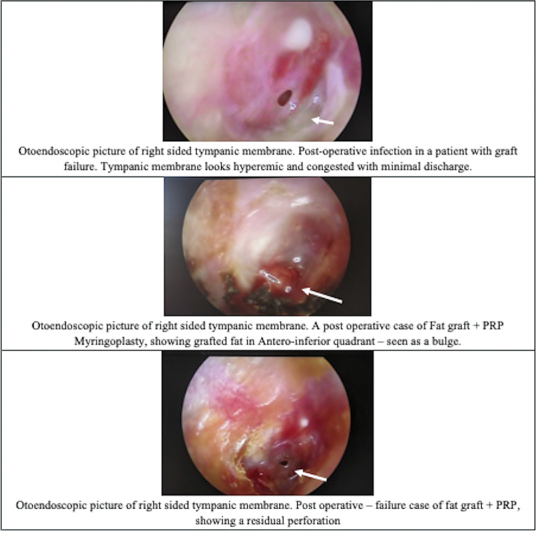
Images showing postoperative infection, graft uptake, and postoperative residual perforation.

Fiorino and Barbieri 8 attributed immediate postoperative infection to one of their cases of graft failure. In the present study, 7 (20.5%) patients developed a postoperative ear infection and 6 (85.7%) of them presented failure in graft uptake ( Fig. 2 ).

The procedures in the present study were all performed by different surgeons, including trainees, with different levels of expertise. Freshening the perforation margins is an important step in the procedure. Chronic perforations undergo epithelialization and, consequently, rolling of their margins, which must be removed to induce epithelial cell growth. Complete stripping of the margins is a technical skill which comes with experience. The fat harvested should be 2 or 3 times the size of the perforation, because it usually undergoes dehydration and necrosis.

Our observations suggest that adult patients who are candidates for type-1 tympanoplasty, with small or moderate-sized central perforations, will benefit from a fat graft tympanoplasty. In developing countries, the large number of cases of chronic suppurative otitis media often overwhelms the surgical infrastructure available. A day-care procedure such as fat graft tympanoplasty is good as a first choice considering the ease with which the surgery can be performed by surgeons with varied levels of expertise. It is also a good choice in patients with long-standing perforations who have developed sensory neural hearing loss and their only need is an ear which remains dry when using the hearing aid. Platelet-rich plasma increases the chances of success of a fat graft tympanoplasty, more so in patients with moderate central perforations, and also leads to better audiometric outcomes.

Temporalis fascia graft resulted in a success rate of 88% according to a meta-analysis. 4 In the present study, fat graft with PRP resulted in a success rate of 82.6% (19 patients), which implies that the procedure merits consideration, keeping in mind that, if the fat graft tympanoplasty fails, tympanoplasty with a standard temporalis fascia graft can be performed later. In the present study, the surgical procedure was performed by surgeons with various skill levels, ranging from senior residents to senior faculty. Therefore, our results indicate a more general expectation of success and failure than any study performed by a single surgeon, which may not be replicable in real life.

## Conclusion

The convention of using temporalis fascia grafts for all central perforations needs to be revisited, for it requires a large incision and may be time-consuming. Fat graft myringoplasty is an effective technique to repair central perforations with small to moderate sizes, with a success rate ranging from 76 to 84% based on different studies. No manipulation of the annulus or middle-ear elements is attempted, and there is no need to raise a tympanomeatal flap. Due to its diverse healing properties, the use of PRP results in better graft survival and uptake (of up to 100% in small central perforations). Moreover, harvesting fat graft from the ear lobule makes it a short and cost-effective day-care procedure, which may be performed under local anesthesia, with comparable functional and anatomical results.
